# Pathogenic Effects of Impaired Retrieval between the Endoplasmic Reticulum and Golgi Complex

**DOI:** 10.3390/ijms20225614

**Published:** 2019-11-09

**Authors:** Hiroshi Kokubun, Hisayo Jin, Tomohiko Aoe

**Affiliations:** 1 Department of Anesthesiology, Chiba University Graduate School of Medicine, Chiba 260-8670, Japan; 2 Department of Medicine, Pain Center, Chiba Medical Center, Teikyo University, Ichihara 299-0111, Japan

**Keywords:** KDEL receptor, endoplasmic reticulum, chaperone, protein quality control, proteostasis, unfolded protein response, ER stress, COPI, GPCR

## Abstract

Cellular activities, such as growth and secretion, are dependent on correct protein folding and intracellular protein transport. Injury, like ischemia, malnutrition, and invasion of toxic substances, affect the folding environment in the endoplasmic reticulum (ER). The ER senses this information, following which cells adapt their response to varied situations through the unfolded protein response. Activation of the KDEL receptor, resulting from the secretion from the ER of chaperones containing the KDEL sequence, plays an important role in this adaptation. The KDEL receptor was initially shown to be necessary for the retention of KDEL sequence-containing proteins in the ER. However, it has become clear that the activated KDEL receptor also regulates bidirectional transport between the ER and the Golgi complex, as well as from the Golgi to the secretory pathway. In addition, it has been suggested that the signal for KDEL receptor activation may also affect several other cellular activities. In this review, we discuss KDEL receptor-mediated bidirectional transport and signaling and describe disease models and human diseases related to KDEL receptor dysfunction.

## 1. Introduction

**Figure 1:**
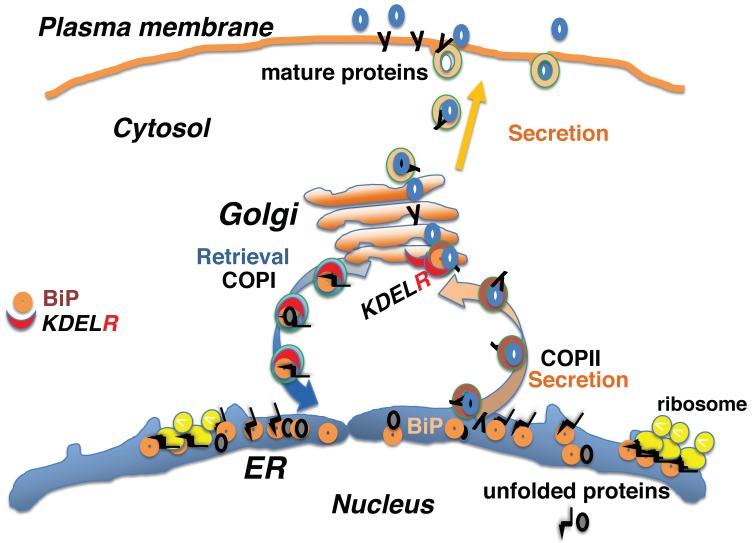
Newly synthesized peptides are incorporated into the endoplasmic reticulum (ER) and transported to the secretory pathway. Interaction with ER-resident chaperones, such as binding immunoglobulin protein (BiP), facilitates the folding of nascent proteins. Mature proteins are then released to the secretory pathway via coat protein II (COPII)-mediated vesicular transport. A fraction of the KDEL sequence-containing chaperones, like BiP, that accompany unfolded proteins are destined for secretion, while KDEL receptors (KDELRs) retrieve escaped chaperones from the *cis*-Golgi and deliver them to the ER through coat protein I (COPI)-mediated vesicular transport.

Cellular activities comprise a series of complex and dynamic processes. Within a cell, organelles undergo continuous and dynamic changes. The localization of intracellular proteins represents their distribution in dynamic equilibrium at a single time point. Proteins move and are distributed throughout the cell. Secreted or membrane-localized proteins are synthesized by membrane-bound ribosomes and are incorporated into the endoplasmic reticulum (ER) membrane through the translocon, which interacts with ER-resident molecular chaperones to facilitate the folding and assembly of multi-subunit peptides [1,2] (Figure 1). Mature proteins are transported to the Golgi complex and subsequently to the cell surface, where they either function as membrane-localized receptors or are secreted from the cell as biologically active molecules. Such protein transport is mediated by various vesicular and tubular transport systems. In addition to protein cargo, membrane components of intracellular organelles are also transported, such that the levels of organellar membranes are balanced through bidirectional transport [3,4,5].

Although ER-resident molecular chaperones, including glucose regulatory protein 94 (GRP94, or heat shock protein 90 β family member 1; HSP90B1), binding immunoglobulin protein (BiP, also referred to as GRP78 or heat shock protein family A (Hsp70) member 5; HSPA5), calreticulin (CALR), and protein disulfide isomerase (PDI), appear to be located solely in the ER lumen, they are widely distributed throughout the cell owing to a dynamic equilibrium achieved through retention and bidirectional transport between organelles. The KDEL-dependent retrieval system was initially identified as a mechanism involved in the retention of ER lumen-resident molecular chaperones containing a carboxyl-terminal Lys-Asp-Glu-Leu (KDEL) sequence within the ER [6]. These chaperones are retained in the ER partly through their interactions with the ER environment, and partly through KDEL receptor-mediated retrieval at the Golgi complex once the chaperones have exited the ER [7]. Proteins containing the KDEL sequence, such as BiP, a major ER-resident molecular chaperone [8] (Figure 1), are recognized by the KDEL receptor in a pH-dependent manner [9]. The acidic environment of the *cis*-Golgi lumen induces a conformational change in the KDEL receptor, allowing it to bind to proteins that contain the KDEL sequence; these proteins are released from the receptor after returning to the ER where the pH is almost neutral [10]. 

The KDEL receptor-mediated retrieval system contributes to protein quality control, which ensures that functional proteins are transported to their correct locations [11,12]. In addition to protein quality control, activation of the KDEL receptor by KDEL sequence-containing proteins also mediates signal transduction [13,14,15,16,17,18,19]. The KDEL receptor is a seven-transmembrane-domain protein that is coupled to heterotrimeric G proteins [19], and emerging evidence has revealed that signaling through the KDEL receptor mediates numerous and important pathophysiological functions, as well as membrane trafficking. Disturbance of KDEL-mediated retrieval may lead to general pathogenic effects, such as enhanced aging and misfolding of essential proteins, leading to various pathological conditions [12,20,21,22,23,24,25,26,27].

## 2. Protein Quality Control in the Early Secretory Pathway Is Mediated by the KDEL Retrieval System

Nascent peptides containing N-terminal signal sequences are bound to ribosomes on the ER membrane and translocate co-translationally into the ER, where they associate with molecular chaperones, such as BiP, to prevent nascent peptide aggregation and degradation [1,2]. Interaction with ER chaperone complexes facilitates the folding of nascent proteins and the assembly of protein complexes. Mature proteins are subsequently released to the secretory pathway by coat protein II (COPII)-mediated vesicular transport [5]. A fraction of the KDEL-containing proteins that are bound to unfolded proteins are destined for secretion; however, they undergo retrograde retrieval from the *cis*-Golgi to the ER via coat protein I (COPI)-mediated vesicular transport [4] (Figure 1).

Misfolded or improperly assembled proteins are bound by ER-resident molecular chaperones and are retained in the ER as unfolded proteins. The T-cell receptor (TCR or TR) multisubunit (α, β, εγ, εδ, and ζζ) complex is assembled in the ER and subsequently delivered to the secretory pathway [28]. Unassembled subunits are associated with ER chaperones. When KDEL receptors become saturated in response to overexpression of KDEL-tagged secretory proteins, unassembled TCRα molecules bound to BiP are not retrieved by KDEL receptors; instead, unassembled TCRα peptides evade quality control in the early secretory pathway and are delivered to the cell surface. This indicates that several KDEL sequence-containing proteins accompanying unfolded proteins, such as TCRα, are secreted from the ER, but are then retrieved through KDEL receptors, a phenomenon that has been observed in a heterologous expression system in cultured cells [11]. 

## 3. Mis-Sorting of KDEL Sequence-Containing Proteins in the Early Secretory System In Vivo 

The in vivo significance of the retrieval of KDEL-containing proteins by KDEL receptors in protein quality control was confirmed in knock-in mice expressing a mutant form of BiP that lacked the KDEL retrieval sequence [21]. Hemagglutinin (HA)-tagged mutant BiP protein was found to be primarily distributed in the ER, as identified by immunofluorescence in embryonic fibroblasts derived from homozygous *Bip* mutant mice. A pulse-chase labeling experiment with [^35^S]-methionine revealed that one-third of the newly synthesized mutant BiP protein was secreted into the culture medium at resting state. Tunicamycin treatment disrupts protein glycosylation in the ER, leading to ER stress [29]. Tunicamycin treatment was shown to enhance the expression of both the mutant and wild-type BiP protein, leading to the extracellular secretion of both BiP forms. These data demonstrate that ER lumen-resident chaperones are localized to the ER largely through interactions with ER membrane proteins and the ER matrix, while a fraction of the chaperones are exported from the ER to the Golgi and retrieved by the KDEL receptor, which is a saturable process.

**Figure 2:**
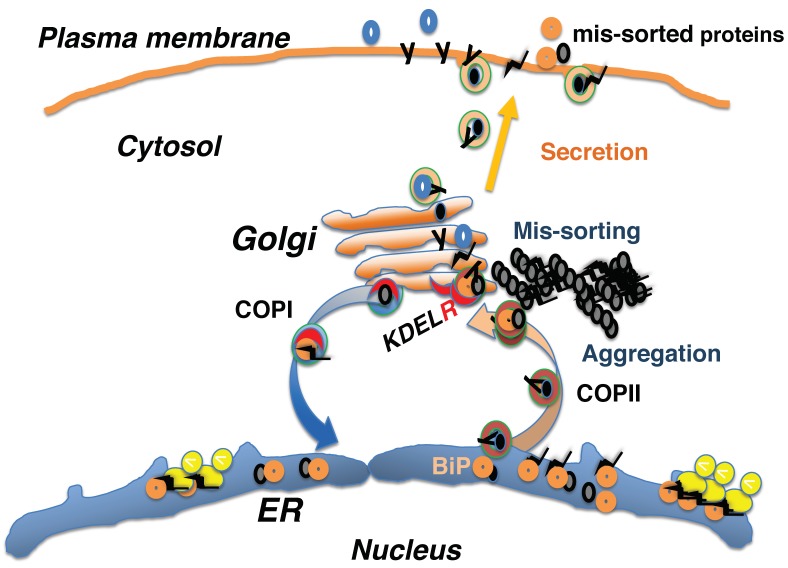
Some proteins, such as SP-C and reelin, require BiP retrieval by the KDEL receptor (KDELR) for proper folding in the early secretory pathway. Impaired retrieval by the KDEL receptor may result in defective proteostasis networks that affect proper protein folding and degradation, leading to protein aggregation. Mis-sorting leads to the presence of immature and KDEL sequencing-containing proteins, such as BiP, on the plasma membrane, where they may perform atypical functions.

BiP is highly expressed in tumor cells [30]. Some of these BiP proteins have escaped the retrieval system and have been transported to the plasma membrane. Here, they are suggested to be involved in cell surface signaling [31]. BiP is also expressed in synovial cells of rheumatoid arthritis patients. BiP transported to the cell surface may be involved in the development of rheumatism through modulation of signal transduction [32]. In addition, cell surface-localized BiP may be recognized as an antigen by T-cells, which may mediate several autoimmune disorders [33] (Figure 2).

Although BiP is essential for cell viability, deletion of its KDEL retrieval sequence is dispensable, at least in a single cell. Embryonic fibroblasts derived from homozygous *Bip* mutant embryos are viable and can be passaged [21]. However, although homozygous *Bip* mutant mice are born at Mendelian ratios, they all die on the first day postpartum. Their breathing is impaired owing to the development of neonatal respiratory distress syndrome that results from impaired pulmonary surfactant secretion. Pulmonary surfactant, composed of phospholipids and pulmonary surfactant protein (SP)-A, -B, -C, and -D, reduces alveolar surface tension to allow spontaneous physiological respiration. Pulmonary surfactant is essential after the transition from the embryonic fluid environment to air breathing after birth. Production of SP-C (SFTPC) was decreased in alveolar type II cells derived from *BiP* mutant neonates, whereas its transcription was maintained. The SP-C precursor contains the BRICHOS domain that prevents amyloid formation [34], and this domain is also found in proteins of the amyloidogenic BRI family that cause neurodegenerative diseases [35]. Several mutations in the *SP-C* gene have been reported to induce protein aggregation and ER stress in the lung [36,37]. C/EBP homologous protein (CHOP), also known as DNA damage inducible transcript 3 (DDIT3), is a transcription factor that induces cell death during ER stress [38,39]. The lungs of *BiP* mutants express high levels of CHOP, suggesting that neonatal respiratory distress syndrome results not only from the loss of pulmonary surfactant function, but also from the accumulation of misfolded surfactant proteins in the ER, which induces ER stress [21,40]. 

Homozygous *BiP* mutant neonates die soon after birth. Although they move and respond to painful stimuli, they are significantly smaller than their wild-type siblings. Furthermore, the *BiP* mutant brain is small and displays disordered neuronal layer formation in the cerebral cortex and cerebellum, as seen in reeler mutant mice [41] that carry a deletion of the reelin (*Reln*) gene. Reelin is a large glycoprotein that guides neuronal migration in the embryonic brain through binding to the very low-density lipoprotein receptor (VLDLR) and apolipoprotein E receptor type 2 (ApoER2), also referred to as LDL receptor-related protein 8 (LRP8), on cortical neurons [42]. Reelin is secreted by neurosecretory Cajal–Retzius cells, which express *Reln* mRNA at a normal level in the *BiP* mutant neonate, but do not produce the reelin protein [22]. 

These observations indicate that some proteins, such as SP-C and reelin, require BiP retrieval by the KDEL receptor for proper folding in the early secretory pathway (Figure 2). 

## 4. Impairment of Retrieval by the KDEL Receptor Causes Myeloproliferative Neoplasms

The significance of KDEL receptor-mediated retrieval for protein folding was confirmed through a different luminal ER chaperone, CALR. Genetic impairment of *CALR* gene has been identified in myeloproliferative neoplasms (MPNs) [26,27]. Approximately 50% to 60% of patients presenting with MPNs carry somatic mutations in the *Janus kinase 2* gene (*JAK2*), leading to constitutive, oncogenic activation of the JAK2-STAT signaling pathway. Mutations in the *CALR* gene were identified in a subset of MPN patients with nonmutated *JAK2* [43]; all the mutations lie in exon 9 and disrupt the carboxy-terminal KDEL sequence of CALR. The steady-state distribution of the mutant CALR protein missing the KDEL sequence appears to be concentrated in the ER, similar to that observed for the mutant BiP form lacking the KDEL sequence [26,27]. However, an accumulation of mutant CALR in the ER-Golgi intermediate compartment was observed in experiments that used embryonic fibroblasts derived from CALR-deficient mice [44]. When the thrombopoietin receptor (TpoR) and mutant CALR were co-expressed, the TpoR remained associated with the mutant CALR protein and Endo H-sensitive (the immature, high mannose form). In contrast, co-expression of the TpoR and wild-type CALR promoted the transition of the immature receptor to the mature, Endo H-resistant form. The mutant CALR protein associates only with the immature, Endo H-sensitive TpoR [44]. Retrieval of the TpoR associated with CALR seems to be required for its proper folding. The immature TpoR may escape quality control in the early secretory pathway and mislocalize to the cell surface. The immature TpoR associated with mutant CALR may exhibit aberrant conformations in the signal-transducing cytoplasmic region, leading to constitutive, oncogenic activation of the JAK2-STAT signaling pathway [44,45,46]. These results suggest that the TpoR may be another example of a requirement for the retrieval of KDEL sequence-containing chaperones by the KDEL receptor for correct protein folding in the early secretory pathway.

## 5. Defective Proteostasis Associated with the Retrieval Pathway Leads to Neurodegeneration

Proteins that remain unfolded are assumed not to be delivered from the early secretory pathway and be degraded by the ubiquitin proteasome system. Alternatively, they may be sequestered as intracellular and extracellular aggregates, leading to stress responses, such as the unfolded protein response (UPR), in the ER [47,48] (Figure 2) and the heat shock response in the cytosol [49,50]. These integrated stress responses result in the production of chaperone proteins, like BiP and heat shock proteins, and increase the capacity of the proteostasis network [51,52]. Failure of these processes can lead to cellular dysfunction and cell death, resulting in various human disorders, such as retinitis pigmentosa [53], early-onset cataracts [54], amyotrophic lateral sclerosis [55], Huntington’s disease [56], Charcot–Marie–Tooth disease type 2L [57], and cardiovascular disease [58]. Most neurodegenerative diseases occur sporadically in middle-aged-to-elderly people, presumably because the capacity of the proteostasis network decreases with age [25,52,59]. In neurodegenerative diseases, protein aggregations are characteristically observed inside and outside neuronal cells [60]. Protein aggregates consisting of α-synuclein, called Lewy bodies, are found in the brains of Parkinson’s disease patients [61], while mutations in α-synuclein and parkin, an E3-ubiquitin ligase involved in proteasomal degradation, have been identified in patients with familial forms of this disease [62]. In Alzheimer’s disease, intracellular neurofibrillary tangles consisting of insoluble tau protein, as well as extracellular senile plaques composed of amyloid-β, are observed [63].

Heterozygous *Bip* mutant mice express both the wild-type and mutant forms of the BiP protein. Although the lifespan of the mutant mice is not significantly different from that of wild-type mice, the mutant is more sensitive to ER stress owing to the impaired capacity of the proteostasis network. With aging, motor impairment developed gradually in some *Bip* mutant mice over one year old. Aggregations of ubiquitinated proteins were found in anterior horn cells of the spinal cord, while neuronal cell death and proliferation of glial cells were also observed [24]. In addition, renal tubular-interstitial lesions also developed with aging [23]. Proteinuria induced by chronic protein overload accelerated these lesions, concomitant with caspase 12 activation and tubular cell apoptosis. 

Organ dysfunction and movement disorders can be detected relatively easily through blood tests and appearance. In contrast, patients with cognitive impairment may appear normal. Memory impairment is an early symptom of neurodegenerative diseases, including Alzheimer’s disease. Cognitive impairment may be difficult to recognize not only in humans, but also in mice. In heterozygous *Bip* mutant mice, a radial maze test revealed that cognitive decline with aging was more pronounced in mutant than in wild-type mice [25]. In mouse models of neurodegeneration, such as Tau [64] and amyloid precursor protein transgenic mice [65], distinct neuronal degeneration occurred because the mice express several-fold copies of the exogenous transgene. The *Bip* mutant mice are heterozygous for deletion of the KDEL sequence [21]. As in patients with sporadic neurodegenerative disease, *Bip* mutant mice may be difficult to identify until they are old. Impaired function of the proteostasis network leads to protein aggregations, which can result in neuronal dysfunction and neurodegeneration [60,66]. Accumulation of ubiquitinated proteins has also been found in the cerebral cortex of aged, *Bip* mutant mice [25].

Injuries, such as proteinuria, exacerbate renal tubular injury in *Bip* mutant mice [23]. In agreement with this, cognitive functions were more significantly affected in *Bip* mutant mice than in wild-type animals following neuronal injury, such as exposure to inhalational anesthetics [25,67]. Although inhalational anesthetics are currently used in clinical practice, recent studies have suggested that they may be neurotoxic under specific conditions. 

Combined, these results suggest that an impaired proteostasis network may contribute to age-related neurodegeneration, and that both the KDEL sequence-containing BiP protein and KDEL receptor play an important role in this process.

## 6. Regulation of Membrane Trafficking and the UPR are Linked through the KDEL Receptor

**Figure 3:**
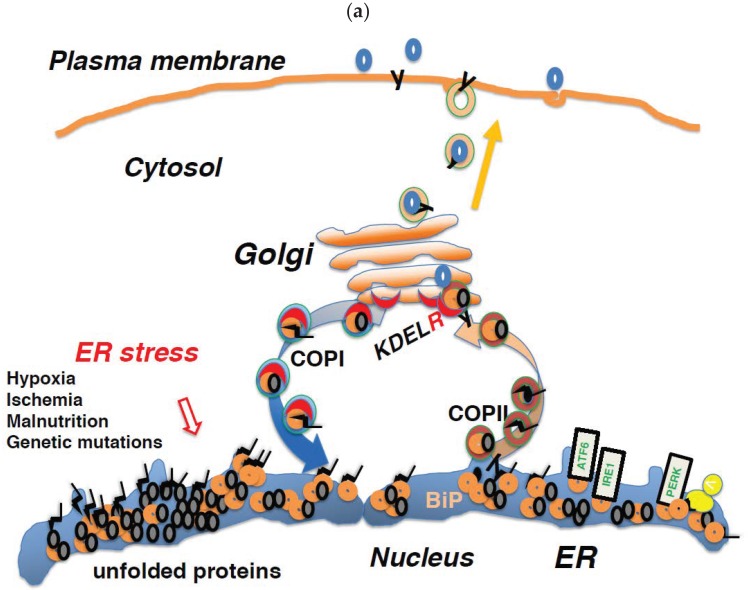

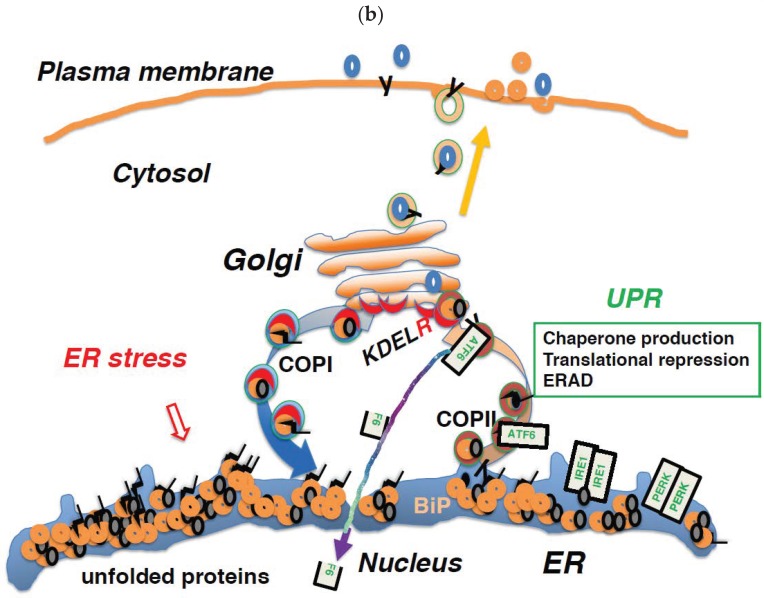
(**a**) Unfolded proteins accumulate in the ER when protein folding is disturbed due to ER stress, which may result from either intrinsic defects (e.g., mutated sequences, missing subunits) or extrinsic injuries (e.g., ischemia, malnutrition, hypoxia, and toxicity). (**b**) BiP dissociates from ATF6, IRE1, and PERK, and associates with unfolded proteins; dissociation from BiP activates ATF6, IRE1, and PERK, and initiates the unfolded protein response (UPR). The cytoplasmic portion of ATF6 is transported to the nucleus; there, it functions as a transcription factor, binding to ER stress response element (ERSE) and promoting the transcription of genes important for the UPR. The UPR increases the ability of cells to deal with increased levels of unfolded proteins through chaperone production, translational repression, and ER-associated protein degradation (ERAD).

The BiP protein is a major ER-resident molecular chaperone [8]. It associates with the ER matrix and ER-resident transmembrane proteins, such as endoplasmic reticulum to nucleus signaling 1 (ERN1, or IRE1), protein kinase RNA (PKR)-like ER kinase (PERK, or eukaryotic translation initiation factor 2 alpha kinase 3; EIF2AK3), and activating transcription factor 6 (ATF6) [68]. These kinases play important roles in the UPR [68]. When protein folding is disturbed due to ER stress resulting from injuries, such as hypoxia, ischemia, malnutrition, and genetic mutations, unfolded proteins accumulate in the ER (Figure 3a). As a result of ER stress, BiP dissociates from these membrane proteins and binds to unfolded proteins, thereby preventing their aggregation. Following its release from BiP, ATF6 is transported to the Golgi complex through COPII-mediated vesicular transport [69]. Then, it is cleaved by site-1 and site-2 proteases [70]. The cytoplasmic portion of ATF6 is transported to the nucleus; there, it functions as a transcription factor, binding to ER stress response element (ERSE) and promoting the transcription of genes important for the UPR, such as *Bip, CHOP*, and *X-box binding protein 1* (*XBP1*). After dissociating from BiP, IRE1 and PERK multimerize and are activated by autophosphorylation [71]. In addition, a mechanism by which unfolded proteins directly activate ERN1 is also assumed to exist [72]. ERN1 is a type I membrane protein containing a serine/threonine kinase domain and an endoribonuclease domain at its cytoplasmic carboxy terminus. Activated ERN1 splices XBP1 mRNA, and the resultant active form of XBP1 mRNA is then translated [70]. The XBP1 protein binds to UPR elements (UPREs) in the transcriptional region of various genes required for the UPR and promotes their transcription [72]. PERK is a serine/threonine kinase that phosphorylates and inactivates eukaryotic translation initiation factor 2A (EIF2A), thereby halting the translation of most proteins [73]. However, the translation of ATF4 is induced. ATF4 promotes *Bip* and *GRP94* transcription through its activity as a transcription factor. In addition, transcription of the *growth-arrest and DNA damage 34* (*GADD34* or *protein phosphatase 1, regulatory subunit 15A; PPP1R15A*) gene is also promoted. GADD34 acts as a phosphatase in conjunction with protein phosphatase 1 (PP1) for dephosphorylation of EIF2A, thereby normalizing protein translation and the UPR [74]. The UPR enhances the ability of cells to deal with increased levels of unfolded proteins through chaperone production, translational repression, and ER-associated protein degradation (Figure 3b).

Professional secretory cells, such as plasma cells and pancreatic β cells, utilize the UPR to expand their capacity for protein folding following a physiological increase in the demand for protein synthesis. Impairment of the UPR results in various disorders related to those secretory cells, such as insufficiency of antibody production and diabetes [68,75]. Homozygous *Bip* mutant mice exhibit neonatal respiratory distress due to defective production of pulmonary surfactant in alveolar type II cells, as well as disordered neuronal migration as a consequence of impaired reelin production in Cajal–Retzius cells [21,22].

In addition to its role in the regulation of membrane trafficking, the KDEL receptor is directly involved in the UPR. The human genome encodes three genes for functional KDEL receptors [7]. Transcription of *KDELR2* and *3*, but not *KDELR1*, is upregulated by XBP1 [76]. During ER stress, this upregulation enhances the expression of KDEL receptors 2 and 3, and increases the capacity for retrieval of KDEL sequence-containing proteins (ER chaperones) according to the levels of misfolded proteins. A point mutation in the *KDELR1* gene was identified in a mutant mouse strain exhibiting a reduced number of naive T cells [18]. The mutant KDEL receptor has a defective association with PP1, which results in prolonged phosphorylation of EIF2A. Because sustained phosphorylation of EIF2A leads to apoptotic processes during the UPR, T cells expressing a mutated form of KDELR1 suffer from a dysregulated ER stress response that can lead to cell death. The KDEL receptor modulates the UPR through its effects on PP1 and EIF2A. The UPR involves the activation of mitogen-activated protein kinases (MAPKs), such as p38 MAPK and c-Jun amino-terminal kinases (JNKs). Activation of the KDEL receptor by ligand binding has been shown to induce the phosphorylation of p38 MAPKs [14].

While an adequate UPR is cytoprotective, a prolonged UPR leads to apoptosis. These results suggest that the KDEL receptor is involved in the UPR through its retrieval ability and modulation of signal transduction.

## 7. Impairment of COPI-Mediated Retrograde Transport Induces ER Stress

Reverse transport from the Golgi to the ER is accomplished by COPI-dependent vesicular transport [4]. The physiological significance of COPI-mediated retrograde transport in higher multicellular organisms was revealed by an analysis of human autoimmune diseases. Several mutations in the *COPA* gene (coding for coatomer protein complex subunit alpha) that affect the same COPA functional domain have been identified in patients with autoimmune diseases characterized by high-titer autoantibodies, inflammatory arthritis, and interstitial lung disease [77]. Mutant COPA proteins elicit abnormal intracellular transport via COPI vesicle formation and impair the binding of proteins that target reverse transport from the Golgi to the ER via COPI-coated vesicles. These COPA variants cannot bind to the dilysine-based motif of ER-resident transmembrane proteins, such as calnexin. Furthermore, expression of the mutated COPA protein has been shown to induce high levels of ER stress in cells, which leads to impaired autophagy. The immunological outcomes associated with COPA mutations may be mediated by ER stress, leading to the production of cytokines that promote the production of TH17 cells that are known to mediate autoimmunity [77]. 

Because activation of the KDEL receptor by KDEL sequence-containing peptides recruits ADP ribosylation factor GTPase activating protein 1 (ARFGAP1) from the cytosol to the membrane, leading to the formation of COPI vesicles [13,78], KDEL receptor impairment may also perturb COPI-mediated retrograde transport. The D193N mutant form of the KDEL receptor is defective in coupling to the G_S_ protein and cAMP-dependent protein kinase A (PKA or protein kinase cAMP-activated catalytic subunit alpha; PRKACA); consequently, it is also defective in subsequent COPI-mediated retrograde transport [3]. The mutant receptor is retained in the Golgi even after ligand binding, and both the retrieval of KDEL-containing proteins and COPI-dependent retrograde transport to the ER are impaired. In vivo, transgenic mice expressing the KDEL-D193N mutant receptor can die spontaneously after reaching adulthood owing to the onset of dilated cardiomyopathy [20]. Moreover, ultrastructural analyses showed an expanded sarcoplasmic reticulum and protein aggregates in cardiomyocytes and these cells were sensitive to ER stress when treated with tunicamycin. Ubiquitinated protein aggregates, enhanced CHOP expression, and apoptosis were also observed in the mutant hearts. 

**Figure 4:**
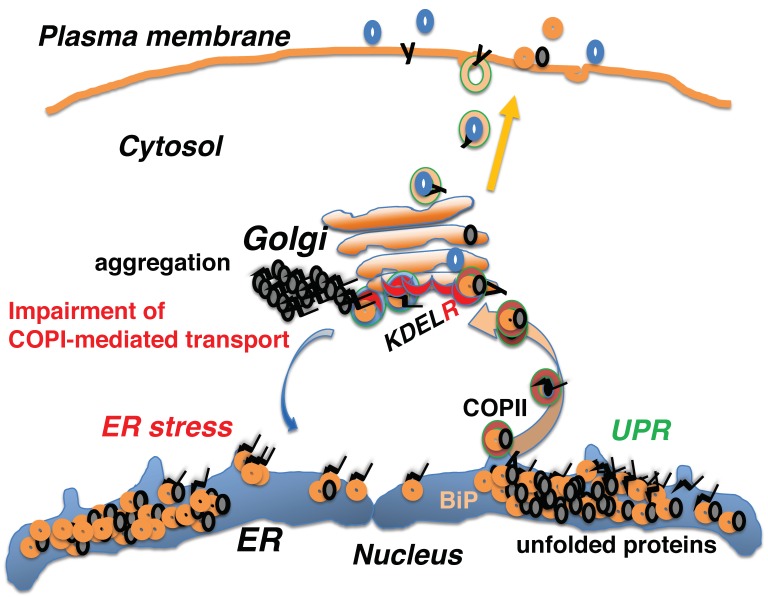
Dysfunction of COPI-mediated transport from the Golgi to the ER disrupts protein folding in the early secretory pathway, resulting in the accumulation of misfolded proteins and the subsequent induction of ER stress and the unfolded protein response (UPR).

These observations revealed that dysfunction of COPI-associated vesicular transport from the Golgi to the ER disturbs protein quality control in the early secretory pathway, resulting in the accumulation of misfolded proteins in the ER and leading to ER stress (Figure 4).

## 8. Activation of the KDEL Receptor May Occur Acutely or Constantly

Newly synthesized proteins interact with ER chaperones for folding. A fraction of nascent peptides bound to ER-resident chaperones, such as BiP, are exported from the ER to the Golgi and constantly activate the KDEL receptor for anterograde transport through the Golgi complex and COPI-mediated retrograde transport to the ER [3,17]. 

The ER is a major cellular calcium (Ca^2+^) storage pool. Activation of cell surface receptors, such as the TCR, initiates a Ca^2+^ flux that leads to Ca^2+^ release from the ER through either the inositol 1,4,5-triphosphate receptor (ITPR) [79,80] or ryanodine receptor [76]. Ryanodine receptors are located in the sarcoplasmic membrane of both muscle and cardiac cells, as well as the ER membrane of other cell types, including neuronal cells [81].

Because ER-resident chaperones, like BiP, are Ca^2+^-binding proteins, depletion of Ca^2+^ stores induces a substantial release of KDEL sequence-containing proteins from the ER and leads to the activation of the KDEL receptor at the Golgi [76], which may be acute. A Ca^2+^ pump located at the ER membrane, ATPase sarcoplasmic/endoplasmic reticulum Ca^2+^ transporting 1 (ATP2A1 or SERCA), retrieves Ca^2+^ from the cytosol and pumps it into the ER lumen [82]. Thapsigargin inhibits ATP2A1, which acutely depletes Ca^2+^ contents in the ER via Ca^2+^ release through ITPR [83]. This induces a massive release of KDEL-containing proteins from the ER and activation of KDEL receptors. 

Tunicamycin obstructs the glycosylation of secretory proteins in the ER [29]. Tunicamycin treatment induces ER stress, where unfolded proteins accumulate in the ER due to disrupted folding of newly synthesized secretory proteins [84]. Alternatively, thapsigargin treatment may reflect acute Ca^2+^ mobilization due to the activation of cell surface receptors, such as TCR, that results in the release of Ca^2+^-binding, ER-resident chaperones from the ER, and activation of the KDEL receptor.

## 9. The KDEL Receptor Signaling Pathway Mediates Various Functions

Heterotrimeric G proteins are coupled to various cell surface receptors called GPCRs (G protein-coupled receptors) [85,86]. The KDEL receptor belongs to a group of seven-transmembrane-domain receptors that bind heterotrimeric G proteins, despite its intracellular localization. G proteins consist of three subunits, α, β, and γ. The Gα subunit mediates the binding of G protein complexes to specific GPCRs and functions in several cellular activities [85,86]. The KDEL receptor has been shown to regulate membrane trafficking through G proteins. Activation of the KDEL receptor by KDEL sequence-containing ligands initiates the subsequent activation of Gα_q/11_ proteins. This leads to the activation of Src kinase and cell division cycle 42 (CDC42), which enhances the anterograde traffic through the Golgi complex [17,87]. Ligand recognition by the KDEL receptor also leads to the activation of Gs and PKA [3]. Phosphorylation of serine 209 in the carboxy terminus of the KDEL receptor by PKA allows the KDEL receptor to associate with ARFGAP1 and coatomer proteins, leading to the formation of COPI-coated vesicles for retrograde transport [15,78]. This information indicates that anterograde traffic from the ER through the Golgi complex and retrograde traffic from the Golgi to the ER are balanced, thereby maintaining organelle structures through KDEL receptor-activated G-protein signaling [3].

In addition to regulating membrane trafficking, activation of the KDEL receptor also influences a wide range of signal transduction pathways. Signaling through the KDEL receptor may play an important role in both physiological and pathological processes. Activation of the KDEL receptor enhances protein degradation pathways. The KDEL sequence-containing protein BiP enhances aggresome delivery to autophagosomes, which reduces the anti-myeloma effect of bortezomib, a proteasome inhibitor [88]. Activation of the KDEL receptor also promotes autophagy [89]; for example, the KDELR-D193N mutant cannot signal through Gs-PKA and is deficient in autophagy. The KDEL receptor also affects the lysosomal protein degradation pathway. The KDEL receptor-Gs-PKA signaling pathway involves lysozyme repositioning to the perinuclear area [90]. Consequently, reduced activation of the KDEL receptor may result in defective proteostasis networks that affect proper protein folding and degradation; this can lead to neurodegenerative diseases, as seen in mutant *Bip* knock-in mice that express a mutant BiP with the KDEL sequence deleted [24].

Src family kinases interact with several cytoplasmic and membrane proteins, phosphorylating tyrosine residues in these proteins and modifying their functions. Activation of the KDEL receptor is suggested to result in extracellular matrix (ECM) degradation via Src activation, a process that may also be involved in tumor invasion [91]. Activation of Src by the KDEL receptor may occur at the Golgi, and activated (tyrosine-phosphorylated) Src may then be transported through secretory pathways. Alternatively, the KDEL receptor may translocate to the plasma membrane, where it activates Src kinase, leading to ECM degradation [92]. 

**Figure 5:**
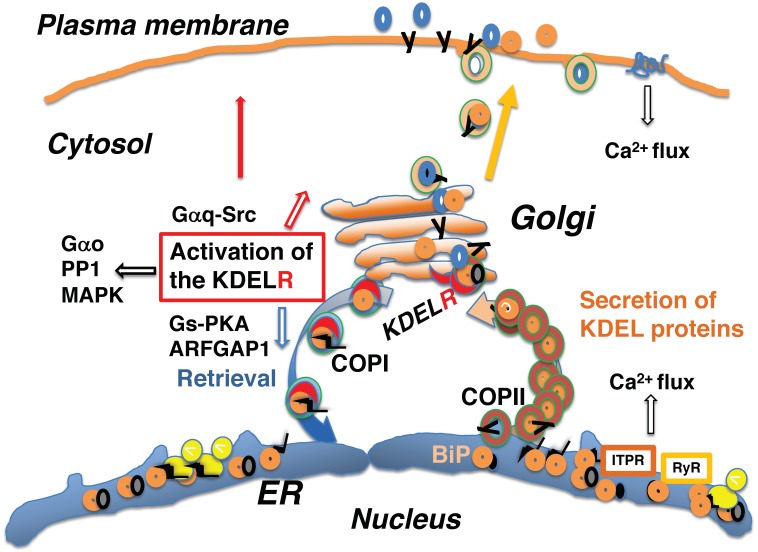
Activation of the KDEL receptor by secretion of endoplasmic reticulum (ER)-resident chaperones or KDEL sequence-containing proteins from the ER regulates intracellular transport, as well as several cellular signaling pathways. Activation of cell surface receptors may initiate Ca^2+^ release from the ER through the inositol 1,4,5-triphosphate receptor (ITPR) or the ryanodine receptor (RyR), inducing secretion of KDEL proteins from the ER.

Gαo is one of the most abundant Gα subunits that associates with cell-surface localized GPCRs, such as adrenergic, dopaminergic, and muscarinic receptors on the plasma membrane. The Gαo subunit is distributed not only along the plasma membrane but also around the Golgi complex. At the Golgi, Gαo associates with the KDEL receptor, the activation of which has been shown to activate Gαo [19]. It is an intriguing possibility that the actions of various cell surface receptors and intracellular KDEL receptors may converge on Gαo, thereby affecting numerous physiological and pathological processes (Figure 5). 

## 10. Conclusions

Cellular activities, such as growth and secretion, are dependent on correct protein folding in the ER. Injury, like ischemia, malnutrition, and toxicity, can affect the ER folding environment. The ER senses this information and the cell adapts to various situations through the UPR. Activation of the KDEL receptor by secretion of ER chaperones or KDEL sequence-containing proteins from the ER may regulate intracellular transport, as well as numerous cellular activities. Future research on the activity of the KDEL receptor may enable control of various cellular functions and contribute to the treatment of several diseases.

## Abbreviations

ER	Endoplasmic reticulum
GRP94	Glucose regulatory protein 94
BiP	Binding immunoglobulin protein
KDEL	Lys-Asp-Glu-Leu
COPII	Coat protein II
COPI	Coat protein I
TCR	T cell antigen receptor
SP	Surfactant protein
CHOP	C/EBP homologous protein
CR	Cajal–Retzius
CALR	Calreticulin
MPNs	Myeloproliferative neoplasms
TpoR	Thrombopoietin receptor
JAK2	Janus kinase 2
UPR	Unfolded protein response
ERN1	Endoplasmic reticulum to nucleus signaling 1
PERK	Protein kinase RNA (PKR)-like ER kinase
ATF6	Activating transcription factor 6
EIF2A	Eukaryotic translation initiation factor 2A
XBP1	X-box-binding protein 1
PP1	Protein phosphatase 1
MAPK	Mitogen-activated protein kinase
JNKs	c-Jun amino-terminal kinases
ARFGAP1	ADP ribosylation factor GTPase activating protein 1
PKA	Protein kinase A
ITPR	Inositol 1,4,5-triphosphate receptor
ATP2A1	ATPase sarcoplasmic/endoplasmic reticulum Ca^2+^ transporting 1
GPCRs	G protein coupled receptors
ECM	Extracellular matrix
